# Splenic Artery Aneurysm With Arterioportal Fistula: An Unusual Cause of Portal Hypertension

**DOI:** 10.7759/cureus.96895

**Published:** 2025-11-15

**Authors:** Riddhima Dubhashi, Suresh Kumar, Sankar Subramaniam

**Affiliations:** 1 Surgical Gastroenterology, Sri Ramachandra Institute of Higher Education and Research, Chennai, IND

**Keywords:** arterioportal fistula, endovascular embolisation, intervention radiology, portal hypertension, splenic artery aneurysm

## Abstract

Splenic artery aneurysms are uncommon vascular lesions. When associated with an arterioportal fistula, they may lead to portal hypertension due to abnormal shunting of arterial blood into the portal venous system. We describe the case of a 28-year-old woman who presented with dull pain in the left upper abdomen. Imaging revealed a splenic artery aneurysm with a communication to the splenic vein. She was managed successfully with endovascular embolisation, which completely excluded the aneurysm and closed the fistula. Early diagnosis and timely intervention are important to prevent rupture or progressive portal hypertension.

## Introduction

Splenic artery aneurysm (SAA) is the most frequent visceral artery aneurysm, accounting for almost two-thirds of all visceral aneurysms [1]. It is seen more often in women and may be associated with pregnancy, portal hypertension, or connective-tissue disorders. Most aneurysms are discovered incidentally, but complications such as rupture or fistula formation can be life-threatening [2]. Arterioportal fistula (APF) between the splenic artery and splenic vein is a rare but important vascular anomaly that may occur spontaneously or secondary to adjacent pathology. The underlying mechanism is usually erosion of an arterial wall into a contiguous venous structure, most often in the setting of a splenic artery aneurysm, trauma, pancreatitis, or previous surgical or interventional procedures [1,2]. The resulting communication allows high-pressure arterial blood to enter the low-pressure portal venous system, producing a left-to-right shunt and a hyperdynamic portal circulation [3]. Over time, this can lead to progressive portal hypertension with splenomegaly, variceal formation, gastrointestinal bleeding, or even cardiac overload in high-flow fistulas [4,5].

Timely diagnosis is essential because untreated fistulas may cause recurrent haemorrhage or progressive hepatic decompensation. Imaging modalities such as Doppler ultrasonography, CT angiography, and digital subtraction angiography help delineate the vascular anatomy and guide therapy [6]. Management options include endovascular interventions, such as selective coil embolisation, vascular plug occlusion, or covered stent placement, as first-line modalities in stable patients, whereas open surgical repair or splenectomy is reserved for those with failed endovascular therapy, rupture, or complex anatomy not amenable to percutaneous approaches [7]. We present a case of splenic artery aneurysm with a splenic artery-splenic vein fistula that was treated successfully by endovascular embolisation.

## Case presentation

A 28-year-old female patient presented with dull, continuous pain in the left upper abdomen for one week. There was no history of trauma, fever, vomiting, or gastrointestinal bleeding. She had not undergone any prior abdominal surgery.

**Figure 1:**
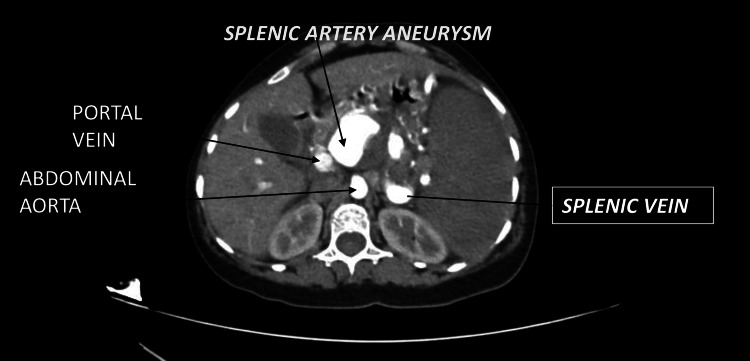
Contrast-enhanced CT Abdomen arterial phase showing splenic artery aneurysm with enhancement of the splenic vein and portal vein in the arterial phase suggestive of a fistula.

**Figure 2:**
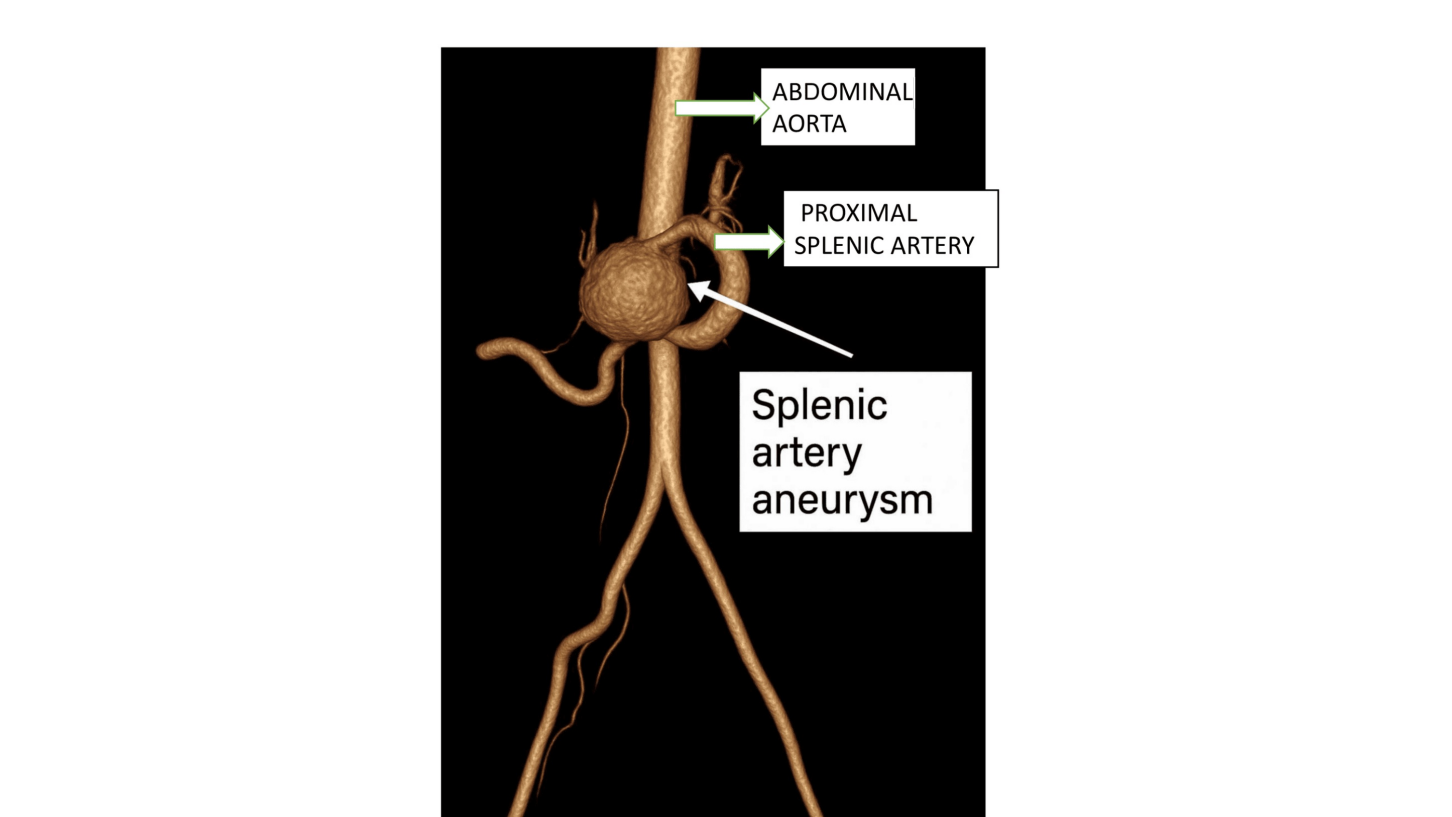
Three-dimensional reconstruction showing a dilated splenic artery aneurysm with the proximal splenic artery and the abdominal aorta.

On examination, she was haemodynamically stable. Mild tenderness was noted in the left upper quadrant without organomegaly. Routine laboratory tests, including complete blood count and liver function tests, were within normal limits (Table 1). Contrast-enhanced CT revealed a 4.1 × 3.9 × 3.6 cm contrast-enhancing aneurysm near the celiac bifurcation, likely from the proximal splenic artery (Figure 1). The distal artery showed filling defects consistent with thrombosis. Early opacification of the splenic and portal veins in the arterial phase indicated a splenic artery-splenic vein fistula. The spleen showed patchy devascularisation. Figure 2 shows a three-dimensional reconstructed image of the aneurysm.

**Table 1:** Laboratory values on admission AST: aspartate aminotransferase; ALT: alanine transaminase; SGOT: serum glutamic-oxaloacetic transaminase; SGPT: serum glutamate pyruvate transaminase

Parameter	Patient Value	Reference Range
Hemoglobin	12.8 g/dL	12–16 g/dL
Total Leukocyte Count	6,700 /µL	4,000–11,000 /µL
Platelet Count	2.5 × 10⁵ /µL	1.5–4.5 × 10⁵ /µL
Total Bilirubin	0.8 mg/dL	0.2–1.2 mg/dL
Direct Bilirubin	0.2 mg/dL	<0.3 mg/dL
AST (SGOT)	26 U/L	10–40 U/L
ALT (SGPT)	22 U/L	10–45 U/L
Alkaline Phosphatase	96 U/L	40–129 U/L
Total Protein	7.0 g/dL	6.0–8.0 g/dL
Albumin	4.1 g/dL	3.5–5.0 g/dL
INR	1.0	0.9–1.1

**Figure 3:**
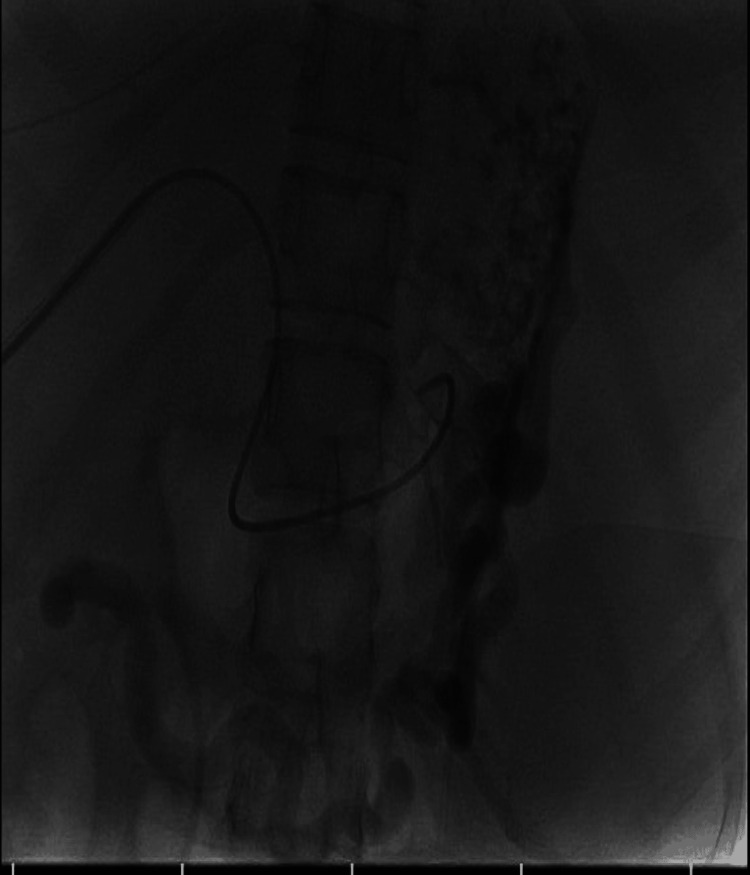
Digital subtraction angiography showing selective catheterisation of the splenic artery prior to embolisation The aneurysm sac is opacified, with early filling of the splenic vein confirming the arterioportal communication.

**Figure 4:**
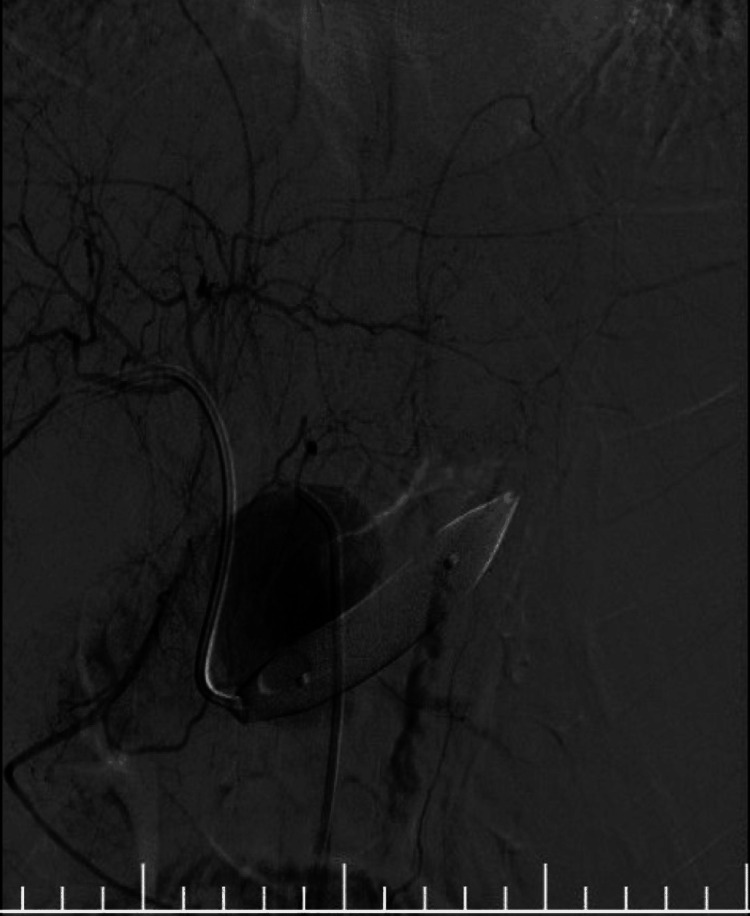
Fluoroscopic image showing balloon occlusion across the fistula site The microcatheter tip is seen within the aneurysm sac during coil deployment.

**Figure 5:**
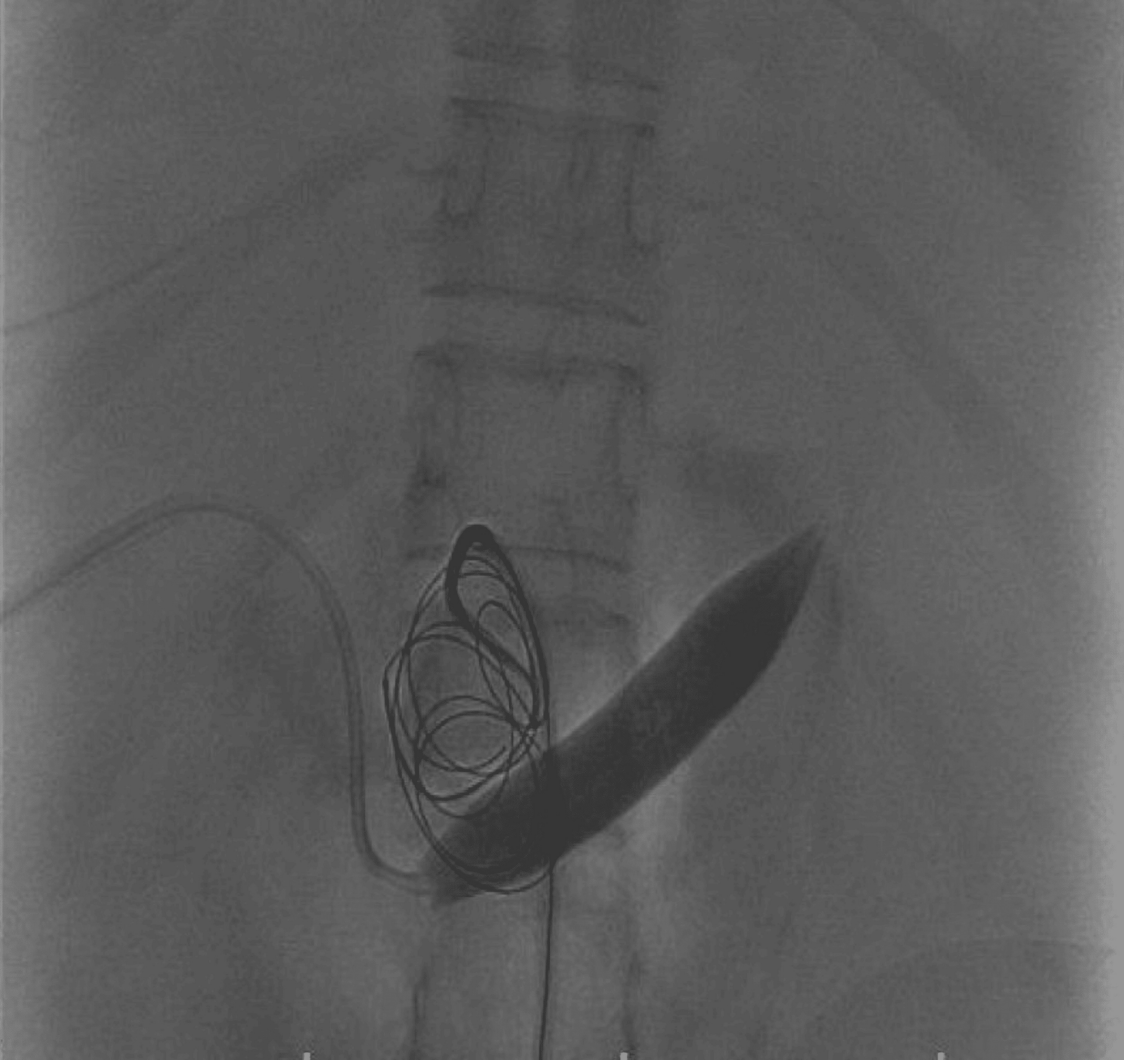
Fluoroscopic image after coil deployment showing multiple coils densely packed within the splenic artery aneurysm sac The balloon remains inflated in the splenic vein to prevent distal embolic migration.

**Figure 6:**
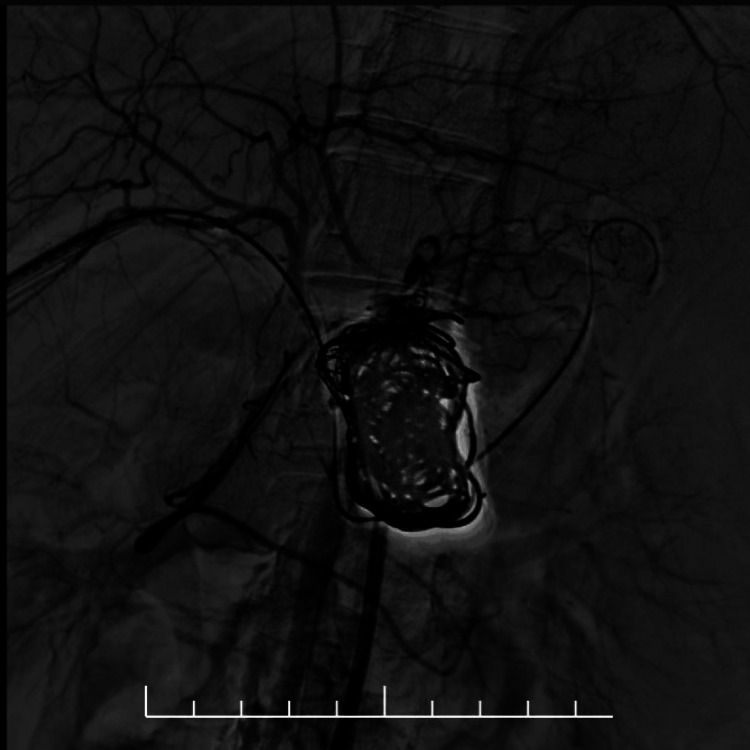
Completion angiogram after combined coil and glue embolisation demonstrating complete exclusion of the splenic artery aneurysm and absence of residual arterioportal shunting No flow is visualised within the aneurysm sac.

After obtaining an interventional radiology consultation, the patient underwent endovascular embolisation. Using the Seldinger technique, right femoral artery access was achieved. A 5-French catheter placed in the celiac artery confirmed a large splenic artery aneurysm with early venous filling (Figure 3). The right segmental branch of the portal vein was punctured, and a 12 × 40 mm balloon was positioned across the fistula in the splenic vein (Figure 4). Eight detachable coils were deployed in the aneurysm, and the proximal stump was sealed with glue. Following multiple coil insertions, a densely packed aneurysm sac was visualised (Figure 5). A completion angiogram demonstrated complete exclusion of the aneurysm and closure of the fistula (Figure 6). The portal venous puncture tract was embolised.

**Figure 7:**
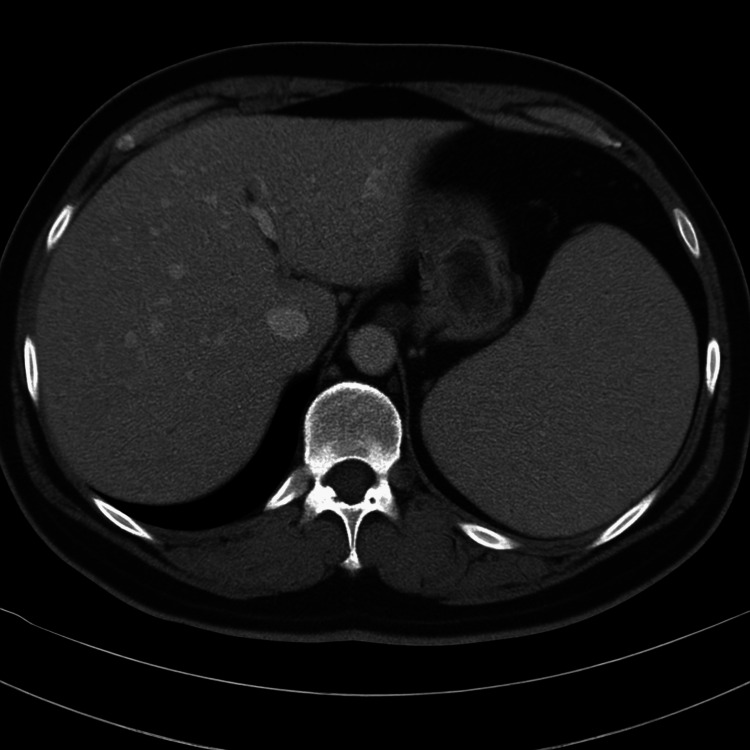
Post-procedure contrast-enhanced axial CT image showing resolution of splenic artery–splenic vein arterioportal fistula with homogeneous enhancement of the spleen and no evidence of infarction

The patient’s recovery was uneventful. She was discharged after two days. Follow-up CT angiography after two weeks confirmed complete thrombosis of the aneurysm and no residual shunt (Figure 7). 

## Discussion

An arterioportal fistula between the splenic artery and the splenic vein is a rare vascular entity that can lead to portal hypertension in the absence of liver disease. Most cases are acquired, commonly following erosion or rupture of a splenic artery aneurysm into the adjacent vein [1,2]. Other causes include trauma, pancreatitis, and previous interventional procedures [3]. The continuous arterial inflow into the portal system raises portal pressure and produces splenomegaly, varices, or hypersplenism [4].

SAAs are the most frequent visceral arterial aneurysms, accounting for nearly 60% of such lesions. They are more common in the female population and in association with portal hypertension, multiparity, or liver transplantation [2,5]. Most are asymptomatic and detected incidentally; however, rupture can be catastrophic, with high maternal and fetal mortality when occurring during pregnancy. Large or symptomatic aneurysms, and those associated with arteriovenous fistulas, require prompt treatment to prevent rupture or high-output portal shunting [1,6].

In this case, the patient presented with features of portal hypertension but had a normal hepatic parenchyma. CT angiography demonstrated a splenic artery aneurysm with a direct communication to the splenic vein, confirming an arterioportal fistula. A Doppler study showed arterialized flow within the splenic vein. Accurate imaging evaluation is essential for defining the anatomy and planning treatment [5].

The main goal of management is to exclude the aneurysm and close the fistulous communication while maintaining splenic perfusion. Endovascular therapy has now become the treatment of choice in stable patients because it offers high success rates with minimal morbidity [6]. Techniques include coil embolisation, vascular plug occlusion, or covered stent placement, depending on the configuration of the aneurysm and the neck of the fistula. Surgical management is reserved for rupture, failed embolisation, or unfavourable anatomy [1,7].

Our patient underwent selective transarterial coil embolisation, achieving complete thrombosis of the aneurysm and closure of the arterioportal communication. Follow-up CT showed homogeneous enhancement of the spleen, no residual filling of the aneurysm, and normalization of the portal vein calibre. Clinically, there was resolution of portal hypertension and splenomegaly.

This case emphasizes that a splenic artery-splenic vein fistula is a rare but reversible cause of portal hypertension. Early recognition with cross-sectional imaging and timely endovascular intervention can prevent complications and preserve splenic function [2,6,7].

## Conclusions

SAA with arterioportal fistula is an exceptionally rare vascular condition and an unusual cause of secondary portal hypertension. CT angiography and digital subtraction angiography play a key role in diagnosis. Endovascular embolisation offers a safe, effective, and minimally invasive therapeutic option with excellent long-term outcomes and minimal morbidity when performed early.
